# Poverty and the Baby

**Published:** 1921-02-26

**Authors:** 


**THE PUBLIC WELL-BEING**—(*continued*).

### Poverty and the Baby.

### A GRAVE FACTOR IN UNEMPLOYMENT.

The poverty caused by the serious spread of unemployment will bring back many problems with which we have been less familiar during the past few years than for many before. Among the sufferers who will most quickly show these effects are the babies, born and unborn. In a working-class home poverty hits straight at the baby. Every attempt is made to shield the tiny thing. The remark of a mother who is trying to feed her husband, herself, and eight children on £1 a week illustrates the attitude of the majority of parents to their children. "So you have to feed ten?" the newspaper interviewer said. "Eight," she replied. "My husband and I can do with scraps." In this queue of sacrifice the baby comes last, but, however unwilling the parents are to deprive it of food and warmth, necessity proves too strong, and in one particular they are helpless—that is, before

**THE PUBLIC WELL-BEING**—(*continued*).

the baby is born. There are many reasons why the birth-rate should have dropped after the war. Children were expensive; homes were scarce; life seemed to have become so cheap that many women were asking if motherhood was worth while; and yet the rate of birth in the past two years has proved so unusually high that uninformed people have been using meaningless phrases about Nature redressing the losses of the war. Probably the explanation is much simpler. Since the period of war rations our diet has steadily improved. If food has been dear, wages have been on the whole high, and for the past two years the workers of the country have probably been better fed than they have ever been before, certainly than they have been for many years. A well-fed woman is not only a better mother, but is a more likely mother than one who is overstrained and undernourished, and we have here probably the explanation both of the astonishingly high birth-rate and the low rate of infant mortality. To those who take long views it is clear that schemes and suggestions for meeting the exigencies of unemployment should be tested by the effect they are likely to have on this question of the nourishment of the working mother.

It is not, however, in the birth-rate, but in the infant-mortality rate that the effects of unemployment will most quickly be seen. At present the mortality rate has been low. Instead of increasing with the birth-rate, it has actually fallen, and the national economy here in wealth and happiness is immeasurable. But to the babies in many homes poverty means death. Directly and indirectly poverty aims its shafts straight at the child in the cradle. Just when nourishment becomes scarce the temperature of the house is lowered, and the lowered vitality of the mother diminishes her ability to make up for the lack of warmth. The child's food obviously deteriorates in quality and amount. The tiny ailments which arise that in normal times would be promptly attended to are neglected or ignored. This fall in what the military man calls *morale* is a far more important factor than is generally recognised. It affects the whole well-being of the child. Meals, besides being short, are uneven and irregular. The whole routine of the home breaks up. Another fact not commonly recognised is the costliness of the most simple things. Cleanliness we demand as being taken for granted, but soap costs money, and to a family declining towards the subsistence line the normal ordinary decencies become things of which account must be taken. These are some of the non-technical ways in which poverty strikes at the baby, but they are by no means unimportant, and a full diagnosis of the trouble will not leave them out of account.

